# Effect of High Salt Concentration (HSC) on Structural, Morphological, and Electrical Characteristics of Chitosan Based Solid Polymer Electrolytes

**DOI:** 10.3390/polym9060187

**Published:** 2017-05-24

**Authors:** Shujahadeen B. Aziz, Omed Gh. Abdullah, Mariwan A. Rasheed, Hameed M. Ahmed

**Affiliations:** 1 Advanced Polymeric Materials Research Laboratory, Department of Physics, College of Science, University of Sulaimani, Sulaymaniyah 46001, Kurdistan Regional Government/Iraq; omed.abdullah@univsul.edu.iq (O.G.A.); hameed.ahmed@univsul.edu.iq (H.M.A.); 2 Development Center for Research and Training (DCRT), University of Human Development, Qrga Street, Sulaymaniyah 46001, Kurdistan Regional Government/Iraq; mariwan.rasheed@univsul.edu.iq

**Keywords:** chitosan polymer electrolyte, degree of crystallinity, SEM, impedance plots, DC and AC conductivity

## Abstract

Chitosan (CS) films doped with sodium triflate (NaTf) were prepared by the solution cast technique. The structural and morphological behaviors of the samples were examined by X-ray diffraction (XRD) and scanning electron microscopy (SEM) techniques. The XRD patterns were deconvoluted to estimate the degree of crystallinity of the samples. The SEM micrograph showed the crystalline structure of the sample contained 50 wt % of NaTf salt. The disappearance of broad peaks of chitosan at 2θ ≈ 21° and 2θ ≈ 32° confirmed the occurrence of ion association at 50 wt % of NaTf salt. In impedance plots, a low frequency spike region and a high frequency semicircle, were distinguishable for low salt concentrations. The highest ambient temperature direct current (DC) electrical conductivity obtained for CS:NaTf was found to be 2.41 × 10^−4^ S/cm for the sample containing 40 wt % of NaTf salt. The role of lattice energy of salts on DC ionic conductivity was also discussed. The temperature dependence of DC conductivity was found to follow the well-known Arrhenius relationship. From the alternating current (AC) conductivity spectra, three distinct regions were recognized for the samples with NaTf salt concentration ranging from 10 wt % to 30 wt %. The plateau region of AC spectra was used to estimate the DC conductivity.

## 1. Introduction

Solid polymer electrolytes (SPEs), have been intensively studied [1], because of their possible application as electrolytes in solid state electrochemical devices such as fuel cells, batteries, super capacitors, sensors, and electrochromic windows [2]. SPEs are usually formed by dissolving alkali metal salts in polar polymers [3]. The advantages of SPEs over conventional liquid electrolytes are high energy density, leak proof, high ionic conductivity, wide electrochemical stability windows, light weight, solvent free condition and easy to design to any shape [4]. Chitosan (CS) polymer, is a derivative of chitin, the most abundant natural amino polysaccharide and is estimated to be produced annually almost as much as cellulose [5,6]. The main commercial sources of chitin are crab and shrimp shells, which are abundantly supplied as waste products of the seafood industry [6]. CS is a polycationic polymer due to the existence of one amino group and two hydroxyl groups in its repeating units [7]. Recently, chitosan has been extensively studied because of its biodegradable, biocompatible, and non-toxic behavior [8]. Some solid polymer electrolytes based on chitosan that have been reported, are chitosan-NH_4_NO_3_-EC [9], chitosan-NH_4_CF_3_SO_3_ [10], chitosan-LiCF_3_SO_3_-EC [11], chitosan-NH_4_I-EC [12], chitosan-PVA-NH_4_NO_3_-EC [13], and chitosan-AgNO_3_ [14]. The conductivity of the above mentioned systems without plasticizers are found to be less than 10^−4^ S/cm. The development of polymeric systems with high ionic conductivity is one of the main goals in polymer electrolyte research, due to their potential application in electrochemical devices [15]. From the fundamental point of view, ionic conduction in polymer electrolytes is poorly understood due to the existence of both crystalline and amorphous phases. It has been reported that the ion conduction takes place primarily in the amorphous phase [16]. To the best of our knowledge, little attention has been paid to polymer electrolytes containing sodium salts. The use of sodium salts in the preparation of polymer electrolytes has several advantages over their lithium counterparts. The softness of sodium based materials makes it easier to achieve and maintain contact with other components in the battery [17].According to the recent review of Vignarooban et al., [18], research and development efforts on sodium-ion batteries are gaining momentum due to their accessibility in abundance at a lower cost than lithium (Li). Many electrolytes used in the state of the art of Li batteries are in general valid also for Na-based batteries due to the chemical similarity between sodium and lithium [19]. Moreover, sodium (Na) based rechargeable batteries are environmentally friendly, non-toxic, and low cost [20]. The main objective of the present work was to investigate the effect of high NaTf salt concentration on structural, morphological, and electrical characteristics of chitosan based solid electrolytes.

## 2. Experimental Details

### 2.1. Materials and Sample Preparation

Solution cast technique was used to prepare chitosan based polymer electrolytes. NaCF_3_SO_3_ (NaTf) (purity 98%, Sigma Aldrich, Warrington, PA, USA) and chitosan (from crab shells; ≥75% deacetylated, Sigma Aldrich, Warrington, PA, USA) were used as the raw materials in this study. One gram of chitosan was dissolved in 100 mL of 1% acetic acid solution. The mixture was stirred continuously with a magnetic stirrer for several hours at room temperature until the chitosan powder was completely dissolved. To these set of solutions 10–50 wt % of NaTf was added separately and the mixtures were stirred continuously until homogeneous solutions were achieved. After casting into various plastic Petri dishes, the solutions were left to dry at room temperature to allow complete evaporation of solvent. The films were kept in desiccators with silica gel desiccant for a further drying process. This procedure yields mechanically stable and free standing samples. Table 1 summarizes the concentration of the prepared solid polymer electrolytes based on chitosan.

### 2.2. Structural and Morphological Analysis

The X-ray diffraction (XRD) measurement was performed to study the nature of complexation between NaTf and chitosan using an X-ray diffractometer (Bruker AXS GmbH, Berlin, Germany). The XRD spectrum was collected at room temperature with operating voltage and current of 40 kV and 40 mA, respectively. The samples were scanned with a beam of monochromatic X-rays with a wavelength of λ = 1.5406 A° and glancing angles of 5° ≤ 2θ ≤ 80° with a step size of 0.1°. A scanning electron micrograph (SEM) was taken to study the morphological appearance using the FEI Quanta 200 FESEM scanning electron microscope (FEI Company, Hillsboro, OR, USA).

### 2.3. Electrical Impedance Spectroscopy (EIS)

Complex impedance spectroscopy is the most commonly used technique for studying the electrical properties of materials and their interface with electronically conducting electrodes. The solid polymer electrolyte (SPE) films were cut into small discs (2 cm diameter) and sandwiched between two stainless steel electrodes under spring pressure. The impedance of the films was measured in the frequency range of 50 Hz to 1000 kHz and at temperatures in the range of 303 K to 413 K, using the HIOKI 3531 Z Hi-tester (No. 1036555, Hioki, Nagano, Japan), which is interfaced to a computer. A LabView 8.2 based software (National Instruments, Austin, TX, USA) was used to control the measurements and calculate the real and imaginary parts of the impedance. *Z′* and *Z″* data were presented as a plot of Nyquist and the bulk resistance was obtained from the intercept of the plot with the real impedance axis. The conductivity was calculated from the following equation [21]:

(1) $$\sigma_{dc}=\left(\frac{1}{R_{b}}\right)\times \left(\frac{t}{A}\right)$$

where, *t* and *A* are the thickness and area of the film, respectively. The real (*Z′*) and imaginary (*Z″*) parts of complex impedance (Z*) was also used for the evaluation of AC conductivity using the following Equations [22,23,24]:

(2) $$\sigma_{ac}=\left[\frac{Z^{\prime }}{{Z^{\prime }}^{2}+{Z^{\prime \prime }}^{2}}\right]\times \left(\frac{t}{A}\right)$$

## 3. Results and Discussion

### 3.1. Structural and Morphological Analysis

**Figure 1:**
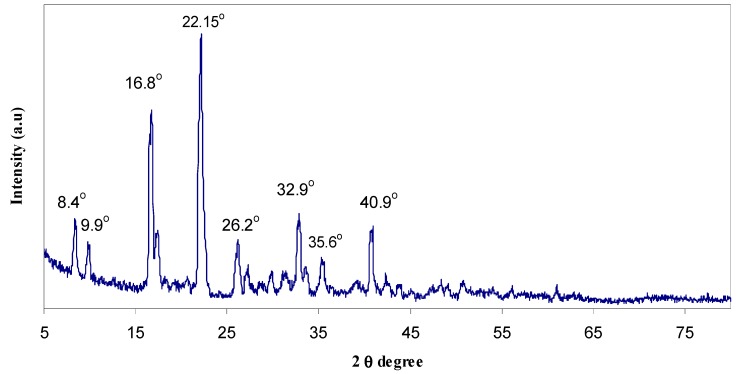
X-ray diffraction (XRD) pattern of pure NaTf salt.

In order to investigate the effect of NaTf on the structure of chitosan-based polymer electrolyte, X-ray diffraction of pure NaCF_3_SO_3_, pure chitosan film and their complexes was performed. Figure 1 shows the X-ray diffraction patterns of pure NaTf. The crystalline peaks of pure NaTf salt can be detected at 2θ = 8.4°, 9.9°, 16.8°, 22.15°, 26.2°, 32.9°, 35.6°, and 40.9°.

**Figure 2:**
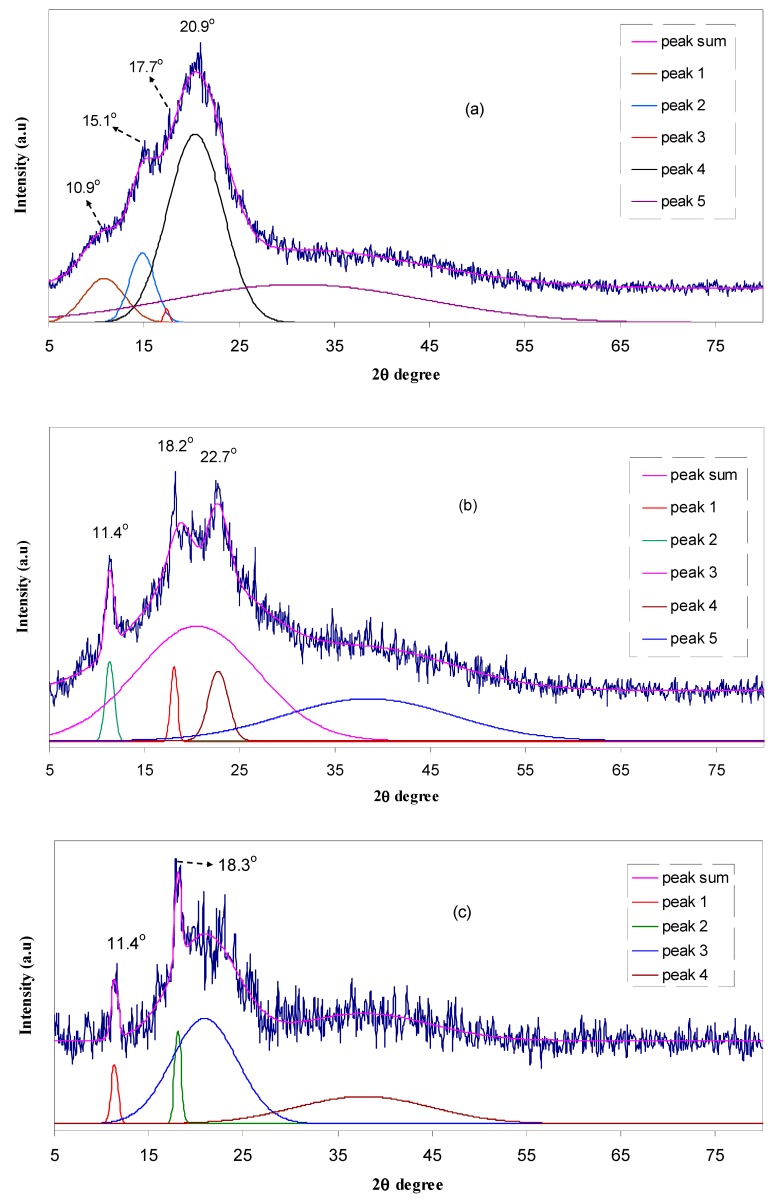

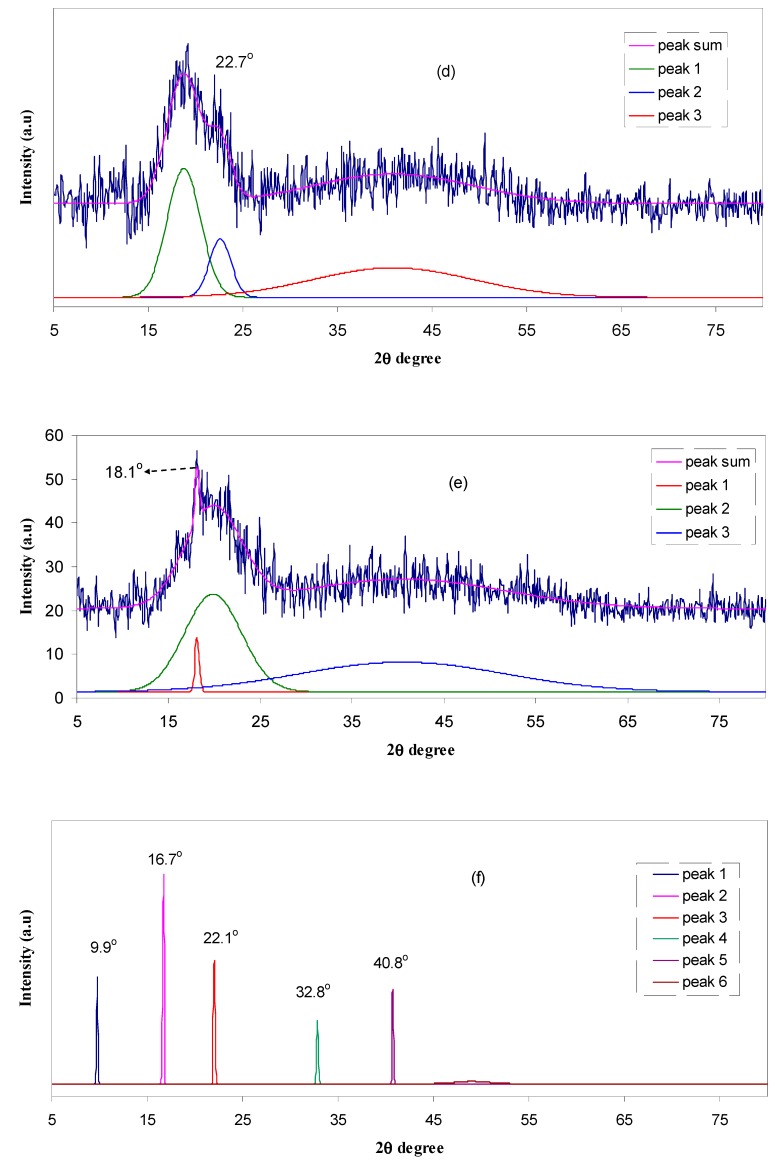
Gaussian fitting of XRD for (**a**) HSCP0; (**b**) HSCP1; (**c**) HSCP2; (**d**) HSCP3; (**e**) HSCP4 and (**f**) HSCP5.

The diffractograms of pure chitosan film and chitosan:NaTf complexes (deconvolution) are exhibited in Figure 2. Pure chitosan is known to possess a semi-crystalline structure with distinguishable peaks at around 2θ = 10.9°, 15.1°, 17.7° and 20.9° [25,26], which are in agreement with the reported values of Wan et al., [27]. These peaks can be ascribed to the average intermolecular distance of the crystalline part of pure chitosan membrane [28]. In our previous work, which was carried out on low NaTf salt concentration (2 wt % to 10 wt %), we observed that the main peaks of chitosan were shifted and split as a result of complex formation between chitosan and NaTf salt [25]. It is clear that the crystalline structure of chitosan is largely retained by intramolecular and intermolecular hydrogen bonding [25,29]. The peak at 2θ = 11.2° is attributed to the reflection plane of (020), while, the peak at around 20.9°, which corresponds to the contribution of two peaks at 18.2° and 22.7° observed in the HSCP1 sample, is related to the reflection planesof (200) and (220) [30]. Another peak appearing near 15.1° is reported to be an indication of the relatively regular crystal lattice (110) of chitosan [30]. It can be noticed that the position of these peaks changes depending on the amount of the incorporated NaTf salt. The sharp peaks at around 11°, 18°, and 22.7° in the doped, HSCP1 to HSCP4, samples are ascribed to the crystalline peaks of chitosan. The broad peaks centered at about 18° and 32° can be attributed to the amorphous nature of chitosan. According to Alves et al., [31] these Gaussian-shaped broad peaks depicted in Figure 2, confirm the predominantly amorphous nature of chitosan solid electrolyte samples. It can be seen that the deconvoluted band appeared at 30° for pure chitosan (Figure 2a) is shifted to about 40° for chitosan incorporated with 10 wt % to 40 wt % of NaTf. As well, the other crystalline peaks of pure chitosan are shifted and their intensities are decreased in the doped samples. The broadening, shifting, and lowering of the relative intensity of chitosan (CS) diffraction peaks on the incorporation of the NaTf salt can be ascribed to the disruption of hydrogen bonding between the polymer chains [23,25,29].

It is clear from Figure 2 that the amorphous area increases with increasing NaTf concentration up to 40 wt % and then the main crystalline peaks of NaTf are found for the sample incorporating 50 wt % of NaTf. To measure the degree of crystallinity (*X_c_*) of membranes, the areas of amorphous and crystalline peaks were calculated. The relative percentage of crystallinities (*X_c_*) were calculated from the following relationship [27,32]

(3) *X_c_* = [*A_c_*/(*A_c_* + *A_a_*)] × 100%

where *A_c_* and *A_a_* are the areas of crystalline and amorphous peaks, respectively. It is interesting to note that the degree of crystallinity is suppressed more effectively upon the addition of more NaTf salt (see Table 2). This can be related to the disruption of the polymer crystalline phase [33]. In our previous works we confirmed through Fourier Transform Infrared (FTIR) spectroscopy that the inter-and intra-molecular hydrogen bonds of chitosan can be weakened or disrupted as a result of complexation occurring between the functional groups of chitosan and the cations of the dopant salt [25,34]. At a high salt content (40 wt %) the system is almost amorphous and only one crystalline peak (peak 1) of chitosan remains. The achieved degree of crystallinity (see Table 2) for pure chitosan (15.1) in the present work was found to be close to the reported value (≈14) of other researchers [27,32,35]. The crystalline peaks appearing in the XRD pattern of HSCP5 are close to the crystalline peaks of NaTf salt (see Figure 1).

**Figure 3:**
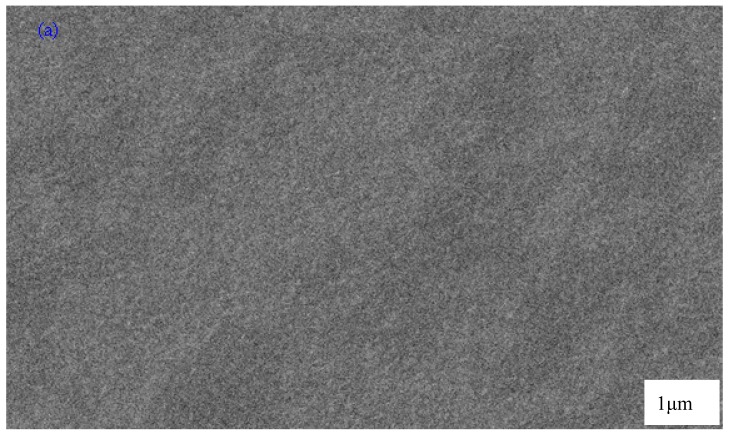

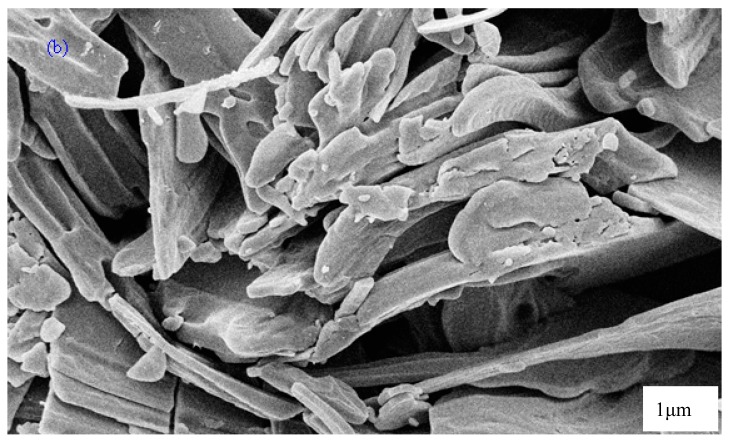
Scanning electron microscopy (SEM) image for (**a**) HSCP 4 and; (**b**) HSCP 5.

To support the XRD results, SEM images were taken for the selected samples. SEM is an efficient technique to explore the surface structure and one of the advantages is that the range of magnification is broad, allowing the area of interest of the sample to be easily focused on by the investigators [36]. The surface morphology was obtained using SEM. SEM provides useful analysis of surface structure and morphology. The nature and morphology of solid polymer electrolyte films are important properties for elucidating their behaviors. Figure 3a,b, show the SEM images of chitosan:NaTf (HSCP 4 and HSCP 5) samples. Morphologically, the HSCP 4 membrane had a uniform surface and it was observed to be smooth and homogenous without any phase separation. When 50 wt % of NaTf salt was added, some crystalline structures appeared to protrude through the surface of the film as shown in Figure 3b. The formation of the crystalline structures can be attributed to ion pair formation, in which the ionic conduction is subtracted [37]. From the SEM results, it is easy to understand that the crystalline peaks obtained for the XRD pattern of the HSCP 5 system (see Figure 2f) are related to the crystalline structures of NaTf salt. These results show that chitosan polymer can dissolve NaTf salt up to 40 wt %. Kadir et al., also used SEM imaging to detect the protruded crystalline structures of salts at high concentrations in the chitosan based solid polymer electrolytes [13,37].

### 3.2. Impedance Analysis

**Figure 4:**
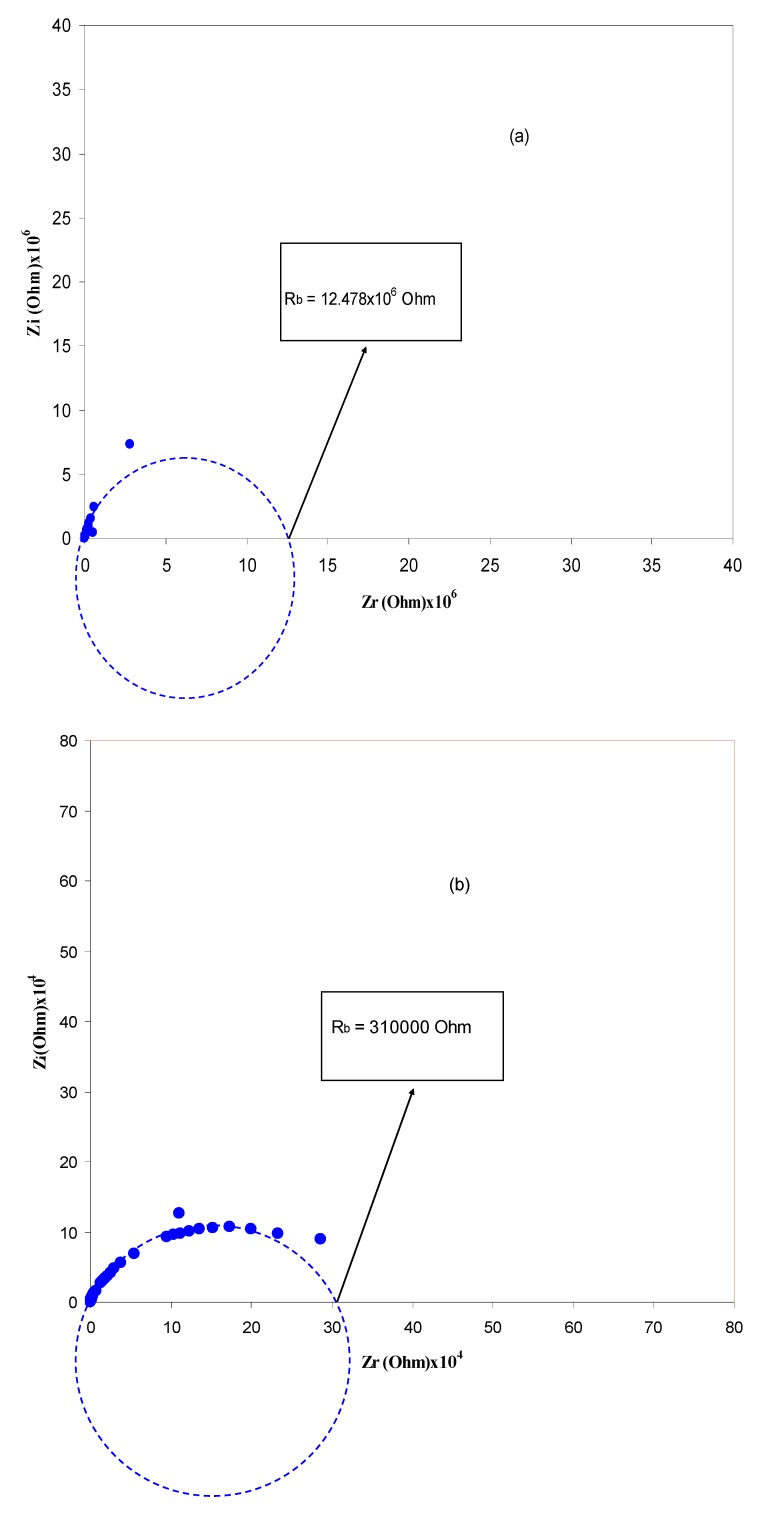

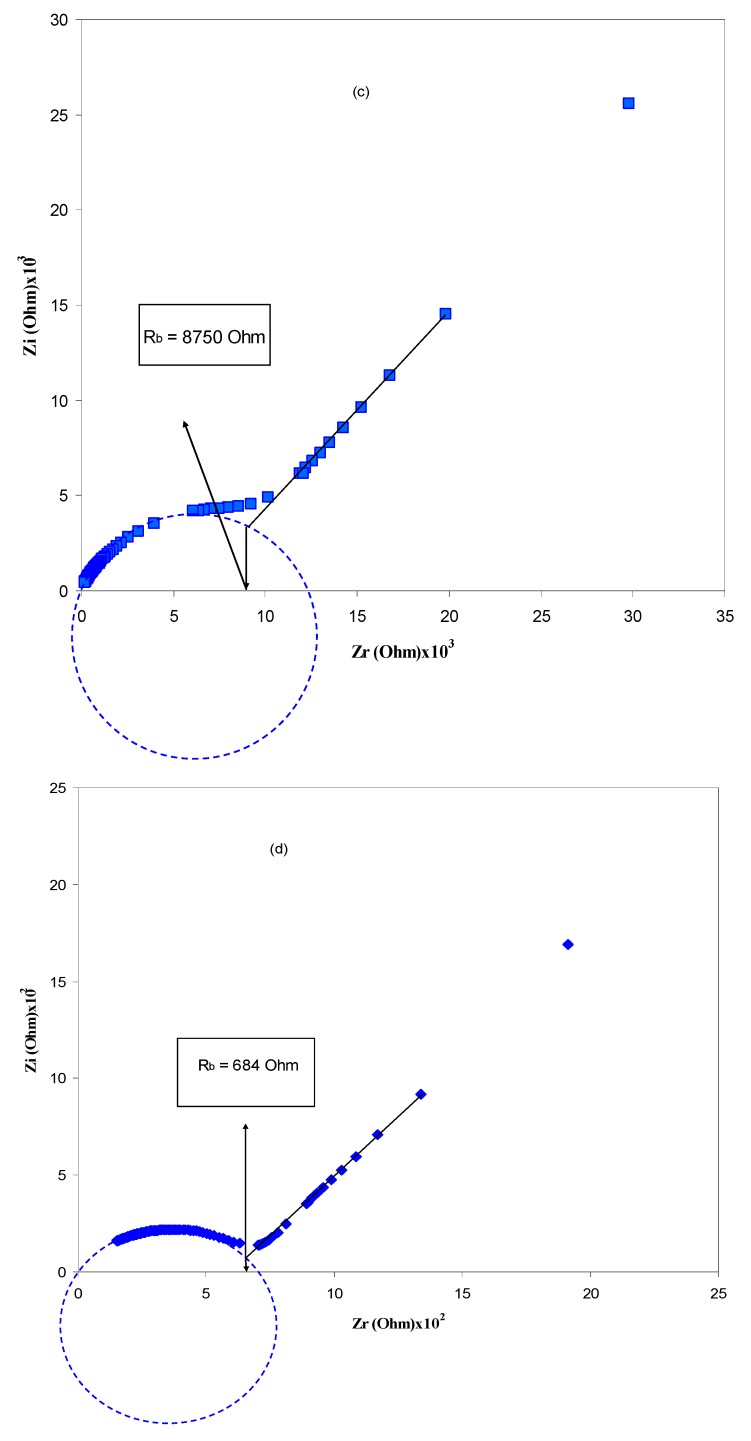

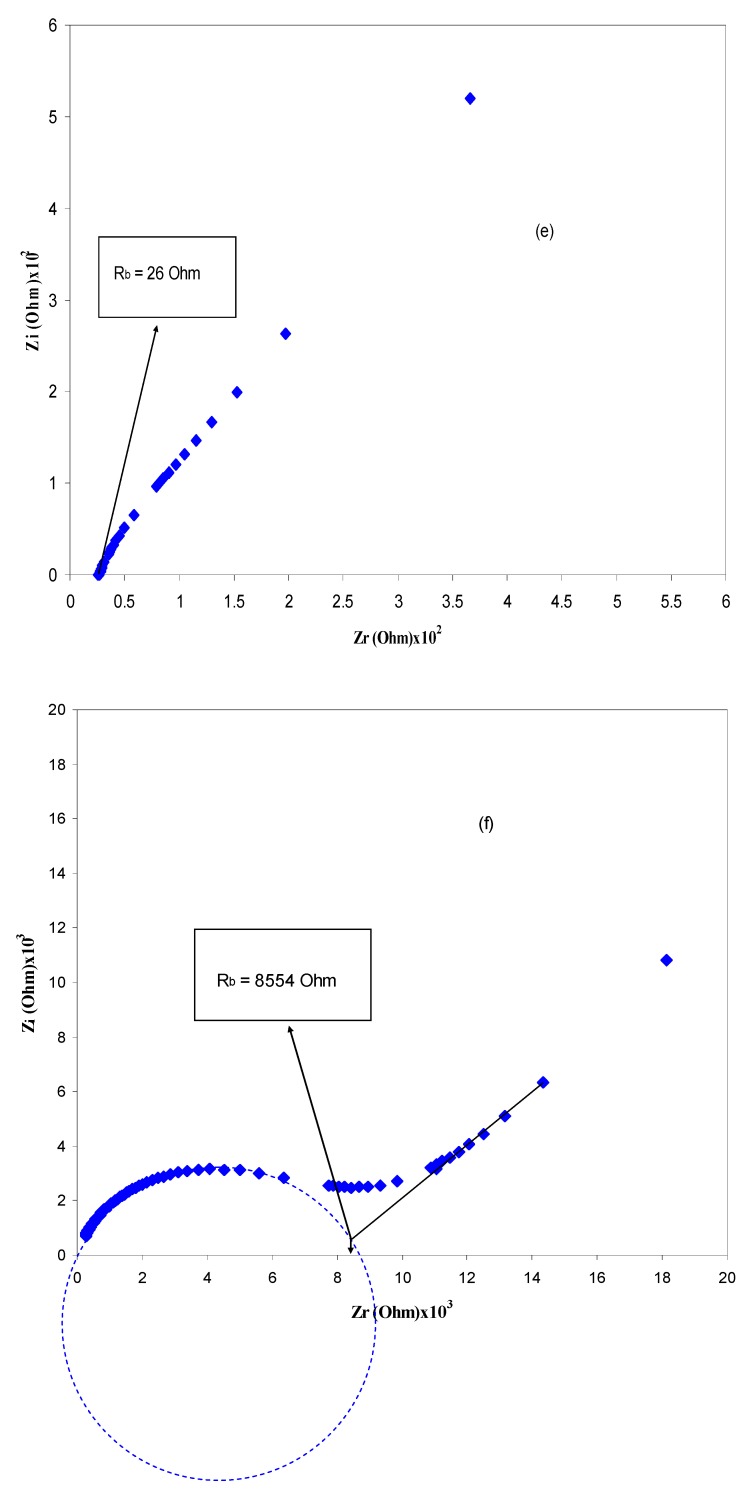
Nyquist plots for (**a**) HSCP 0; (**b**) HSCP 1; (**c**) HSCP 2; (**d**) HSCP 3; (**e**) HSCP 4 and (**f**) HSCP 5.

Electrochemical impedance is a powerful tool to study the electrical properties of electrodes and polymer electrolytes [38]. Electrical impedance plots (i.e., *Z*_i_ vs. *Z*_r_) for all the samples are shown in Figure 4a–f. From Figure 4a–d, it can be seen that the plots show two obvious regions, high frequency semicircle and low frequency spike regions. The spike region occurs as a result of the formation of electric double layer (EDL) capacitances by free charge accumulation at the interface between the solid electrolyte and electrode surfaces [39]. In fact the plots of complex impedance at the low frequency region must show a straight line parallel to the imaginary axis, i.e., the inclination of the straight line should be 90°, but the blocking double-layer capacitance at the blocking electrodes causes this inclination [40,41]. The high frequency region can be used to obtain the bulk resistance (*R_b_*) as shown in Figure 4. From Figure 4a–d, the high frequency semicircular region and low frequency spike can be observed. It is obvious that the high frequency semicircle diameter gradually decreases with increasing salt concentration and almost disappears at 40 wt %. The disappearance of the high frequency semicircular region in the impedance plot leads to the conclusion that the total conductivity is mainly the result of ion migration at higher salt concentration [42].This makes the determination of DC conductivity more difficult because the arc is completely absent (see Figure 4e). In this case, the DC conductivity was determined by extrapolating the polarization “spike” in the complex plane to the intersection with the real impedance as depicted in Figure 4e [43]. The spike tail usually appears in polymer electrolytes at high salt concentration and at high temperatures [13,42]. The appearance of a high frequency semicircle in the HSCP 5 system can be ascribed to ion association at high salt concentration, which in turn decreases the conductivity. When ion association occurs at high salt concentration; the peaks of crystalline NaTf salt may appear as depicted in the XRD pattern of the HSCP 5 system (see Figure 2f). The distinguishable crystalline structures observed in SEM micrograph for the HSCP 5 system (Figure 3b) confirmed the occurrence of ion association. Thus, the electrical properties are strongly supported by the results of XRD and SEM.

### 3.3. DC and AC Conductivity Analysis

It has been well reported that the ionic conductivity of solid polymer electrolytes depends on the number of the charge carriers and their mobility as follows [44]:

(4) *σ* = *Σ n_i_ z_i_ µ_i_*

where *n_i_, z_i_*, and *µ_i_* refer to the number of charge carriers, the ionic charge, and the ionic mobility, respectively. Therefore, according to Equation (4) the DC ionic conductivity can be enhanced by increasing the salt concentration or mobility. Table 3 represents the calculated DC conductivity for all the samples. It is obvious that the DC ionic conductivity is increased with increasing salt concentration up to 40 wt % of NaTf and then drops. Previous study revealed that the dependence of ionic conductivity on the salt concentration may be useful to obtain certain information on polymer-salt interactions and their miscibility [45]. From Table 3 it is clear that with up to 40 wt % of NaTf salt, there is a good compatibility between the chitosan and the added salt, whereas incorporation of more salt causes a drop in DC conductivity. The room temperature DC conductivity of the samples is strongly supported by the results of XRD and SEM. Thus, a good structure-property relationship can be observed from the results of the present work. The appearance of crystalline peaks of NaTf salt at 50 wt % (see Figure 2) is responsible for the drop in DC conductivity. The observed crystalline structures in SEM image at 50 wt % of NaTf salt supports the XRD results. The occurrence of re-association of anions and cations of dissolved salt in polymer electrolytes is expected at high salt concentrations. It has been well established that when a high salt is added to the polymer host, the ions and cations can be close enough to form salt aggregates, which will hinder other free ions from moving and reduce the number of the density of free mobile ions [46]. This phenomenon (salt aggregation) has been reported widely in the literature for chitosan based solid polymer electrolytes incorporated with high salt concentrations [13,37,46,47]. A maximum DC conductivity of 2.41 × 10^−4^ S/cm was achieved in the present work, which is higher than 1.53 × 10^−6^ S/cm for chitosan:europium triflate and 5 × 10^−6^ S/cm for chitosan:lithium triflate (LiCF_3_SO_3_) reported by Silva et al., and Arof et al. respectively [48,49]. However, it is very close to the value of 2.27 × 10^−4^ S/cm reported by Rosli et al., for hexanoyl chitosan/polystyrene-LiCF_3_SO_3_ incorporated with TiO_2_ nanoparticle [50]. The high DC conductivity of chitosan with Na^+^ cation compared to other salts can be explained based on the theory of Hard-Soft-Acid-Base (HSAB) introduced by Ralph Pearson. According to this theory, the larger (e.g., Ag^+^ or Na^+^) and smaller (e.g., Li^+^) cations are considered to be soft and hard, respectively [51]. A strong bond can be formed between a hard cation and a hard anion as well as a weak bond between a soft cation and a hard anion, resulting in a higher DC conductivity. The formation of bonds between cations and anions of the salts can be better understood from the salts’ lattice energy. It was established that the dissociation of inorganic salts in macromolecular solids depends on the lattice energy of salts and the dielectric constant of the host polymer [52]. The lattice energy of NaTf is 650 kJ/mol and is smaller than the lattice energy of LiCF_3_SO_3_, which was found to be 725 kJ/mol [52]. From the above discussion it is clear that in addition to the dielectric constant of the host polymers the lattice energy of the salts significantly affects the conductivity behavior of polymer electrolytes.

**Figure 5:**
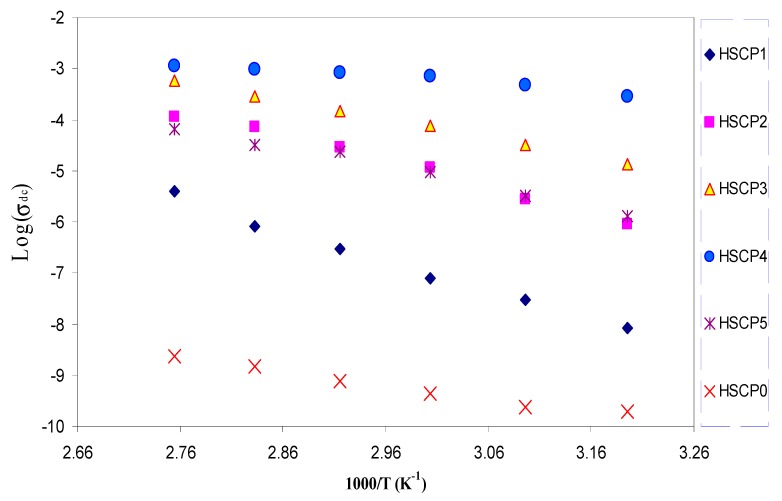
Temperature dependence of DC ionic conductivity for all samples.

**Figure 6:**
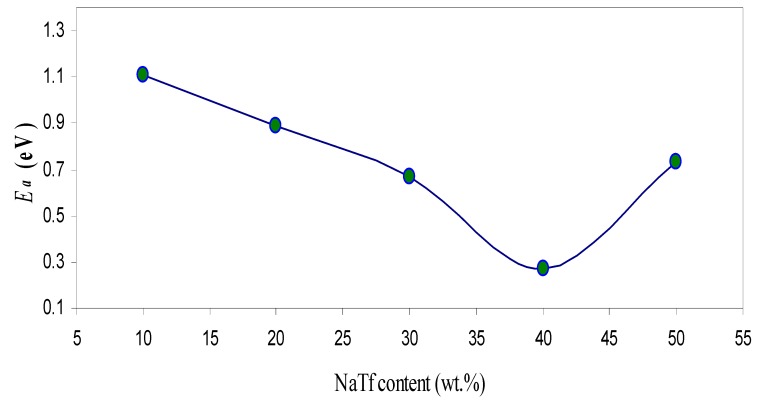
Variation of activation energy (*E_a_*) with salt concentration.

Figure 5 shows the temperature variation of DC conductivity for various salt concentrations. One can see that the DC conductivity is increased almost linearly with increasing temperature. At high temperature, the thermal movement of polymeric chain segments and salt dissociation could be improved. This encourages the ion transport and accordingly causes the ionic conductivity of the polymer salt complex to be raised [25,53]. It is interesting to note that the increase of DC conductivity versus 1000/T is not a rapid process. This reveals that the hopping of mobile ions from one site to another is a thermally activated process [54]. The linear relations observed in all chitosan:NaTf samples highlights that there is no phase transition in the polymer electrolyte [55], i.e., the temperature dependence of ionic conductivity in the temperature range is of the Arrhenius type:

(5) *σ_dc_* = *σ_o_* exp[*−E_a_*/*K_B_T*]

where *σ_o_* is a pre-exponential factor, *E_a_* is the activation energy, *K_B_* is the Boltzmann constant, and *T* is the temperature (K). The calculated *E_a_* value for the highest conducting sample was found to be 0.27 eV. Figure 6 illustrates the activation energy as a function of NaTf salt concentration. One can clearly see that the activation energy has decreased with increasing salt concentration up to 40 wt % and then increased. The activation energy may be considered as an energy barrier, which the ion has to overcome for a successful jump between the sites [21]. It is well known that ionic motion in polymers with high amorphous portions is easier than in polymers with high crystalline portions. The low activation energy of HSCP 4 is related to its high amorphousness (*X_c_*). Kumar et al., [56], reported that crystalline regions in SPEs can hinder the ion movement by blocking the paths to ions. However, the increase of amorphous region results in an increase in the free volume. The increase in free volume would facilitate the motion of ionic carriers. Consequently, the sample with a large amorphous portion exhibits a higher DC conductivity with lower activation energy.

**Figure 7:**
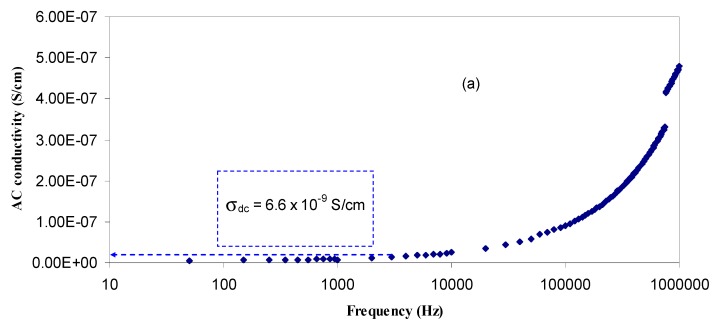

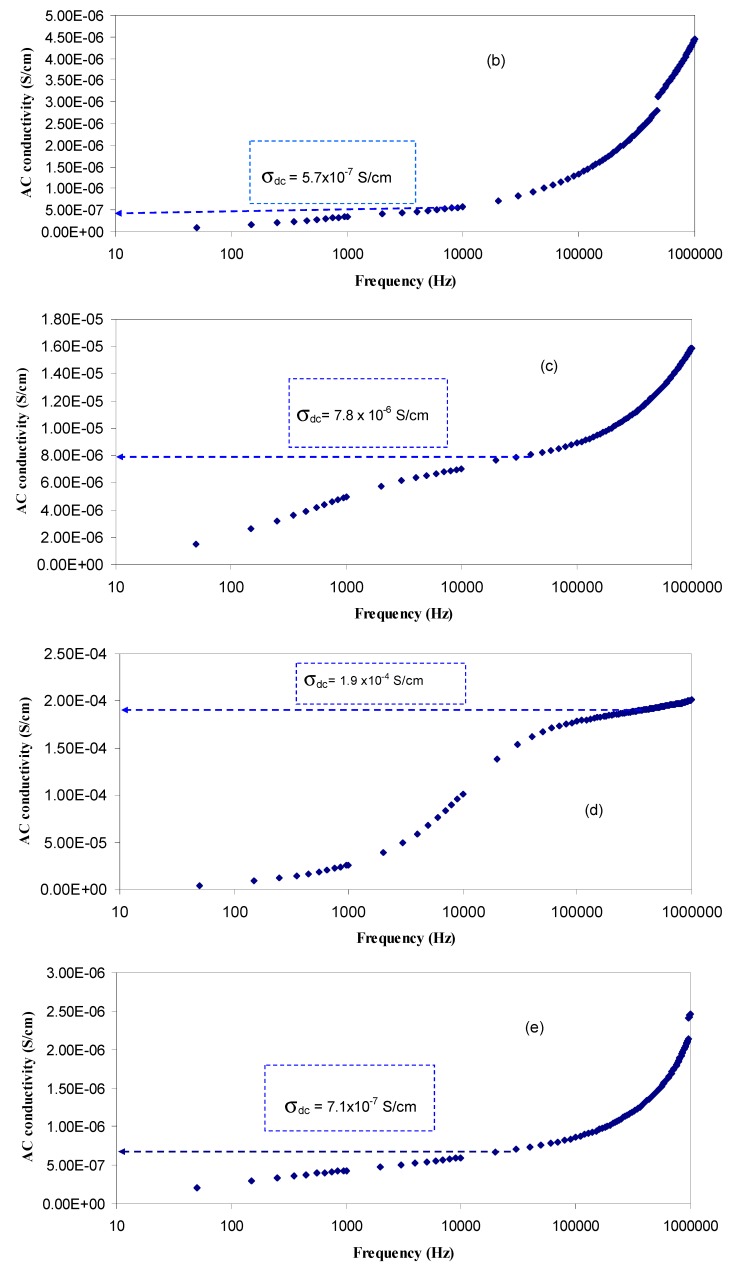
AC conductivity spectra for (**a**) HSCP 1; (**b**) HSCP 2; (**c**) HSCP 3; (**d**) HSCP 4 and (**e**) HSCP 5.

Figure 7 illustrates the AC conductivity spectra for all the doped samples. It is clear that the contribution of the spike region increased with increasing salt concentration. Previous studies established that different electrical phenomena can be seen in AC conductivity spectra, such as, (i) dielectric relaxations (dispersion region), which can be measured usually at high frequencies and low temperature; (ii) phenomena related to transport of charge through the electrolyte, e.g., ionic DC conductivity (plateau region); and (iii) interfacial properties, which dominate the spectra at low frequencies and high temperature (spike region) [22,57]. Therefore, different contribution can be studied from the frequency dependent measurements. The low frequency region (I), which appears as a spike, can be due to electrode–electrolyte interfacial phenomena, i.e., electrode polarization (EP) effect [24]. It can be observed that the contribution of the spike region (I) increases with the increase of salt concentration. From the intermediate frequency region (II), a plateau of AC conductivity is observed. This region corresponds to the DC conductivity [23] and decreases significantly with increasing salt concentration as a result of electrode polarization (EP) enhancement as well as shifts to the higher frequency side. It has been well reported that DC conductivity is related to the presence of free charges in the polymer electrolyte systems. Whereas, AC conductivity belongs to trapped charges in the defect levels that can be activated in the high frequency region [58]. The extrapolation of the plateau region to the y-axis was used to estimate the DC conductivity. The DC conductivity values achieved from the AC conductivity spectra are close to those calculated from the bulk resistance (*R_b_*) (see Table 3).

## 4. Conclusions

Chitosan (CS) films doped with sodium triflate (NaTf) were prepared by the solution cast technique. NaTf was added to CS in different weight ratios ranging from 10 wt % to 50 wt % in steps of 10 wt %. The results of XRD revealed that the amorphous portion increases with increasing NaTf concentration up to 40 wt %. The XRD patterns were deconvoluted to estimate the degree of crystallinity. The smallest degree of crystallinity was obtained for the HSCP4 sample. The crystalline peaks appearing at 50 wt % NaTf were attributed to the ion aggregates that resulted from ion association, occurring at high salt concentrations. The disappearance of broad peaks of chitosan at 2θ ≈ 21° and 2θ ≈ 32° confirms the formation of crystalline domains as a result of ion association at 50 wt % NaTf. The SEM result of HSCP5 proved that ion aggregates are able to leak from the surface. In impedance plots the low frequency spike region and high frequency semicircle were distinguishable for salt concentrations ranging from 10 wt % to 30 wt %. The highest ambient temperature DC electrical conductivity obtained for CS:NaTf was 2.41 × 10^−4^ S/cm for the sample containing 40 wt % NaTf. The temperature dependence of DC conductivity was found to follow the Arrhenius equation. The smallest activation energy was achieved for the sample incorporated with 40 wt % NaTf. The dispersion region in the AC conductivity spectra was found to be distinguishable up to 30 wt % NaTf. The estimated DC conductivity from the extrapolation of the plateau region of the AC spectra was close to those values calculated from the bulk resistance. These results reveal the accuracy of our measurements in the present work.

## Figures and Tables

**Table 1:** Composition of chitosan:NaTf based solid polymer electrolytes.

Sample designation	Chitosan (g)	NaTf (wt %)	NaTf (g)
HSCP 0	1	0	0
HSCP 1	1	10	0.11
HSCP 2	1	20	0.25
HSCP 3	1	30	0.42
HSCP 4	1	40	0.66
HSCP 5	1	50	1

**Table 2:** Degree of crystallinity (*X_c_*) for all the samples.

Sample designation	Degree of crystallinity (*X_c_*)
HSCP 0	15.1
HSCP 1	13.4
HSCP 2	9.7
HSCP 3	7.2
HSCP 4	1.97
HSCP 5	-

**Table 3:** DC ionic conductivity of pure chitosan and chitosan:NaTf complexes at 30 °C.

Sample designation	DC conductivity (S/cm)
HSCP0	1.65 × 10^−10^
HSCP1	4.78 × 10^−9^
HSCP2	5.84 × 10^−7^
HSCP3	8.53 × 10^−6^
HSCP4	2.41 × 10^−4^
HSCP5	7.34 × 10^−7^
